# Prognostic value of seminal vesicle invasion on preoperative multi-parametric magnetic resonance imaging in pathological stage T3b prostate cancer

**DOI:** 10.1038/s41598-020-62808-z

**Published:** 2020-03-30

**Authors:** Jung Kwon Kim, Hak Jong Lee, Sung Il Hwang, Gheeyoung Choe, Sung Kyu Hong

**Affiliations:** 1 Department of Urology, Seoul National University Bundang Hospital, Seongnam, Korea; 2 Department of Radiology, Seoul National University Bundang Hospital, Seongnam, Korea; 3 Department of Pathology, Seoul National University Bundang Hospital, Seongnam, Korea; 4 Department of Urology, Seoul National University College of Medicine, Seoul, Korea

**Keywords:** Cancer imaging, Surgical oncology

## Abstract

We aimed to evaluate the prognostic value of seminal vesicle invasion (SVI) on preoperative multiparametric MRI (mpMRI) in pathological T3b prostate cancer (PCa). We retrospectively reviewed the clinical data of patients who underwent preoperative mpMRI and subsequent radical prostatectomy (RP). A total of 159 patients with pathologic T3b PCa were stratified into two groups based on mpMRI findings (negative vs. positive SVI). A positive SVI was defined as the presence of mpMRI evidence of SVI. In addition, 290 patients with pathologic T3a were also included in this study for further comparative analysis. Fifty-two patients (32.7%) had a positive SVI on preoperative mpMRI. Biochemical recurrence (BCR) occurred in a total of 45 (28.3%) patients, with 25 (23.4%) cases in the negative SVI group and 20 (38.5%) cases in the positive SVI group. Kaplan-Meier survival analysis of the two groups revealed significantly decreased BCR-free survival in the positive SVI group (median, 21 vs. 9 months, log-rank test, P < 0.001). On multivariate Cox regression analysis, pre-biopsy PSA (P = 0.035) and positive SVI on preoperative mpMRI (P = 0.049) were identified as significant predictors of BCR. Upon further comparative analysis with the pathologic T3a group, we also found significant differences among the groups throughout the Kaplan-Meier curve (P < 0.001). Conclusively, the unpredicted (negative) SVI group had a favorable BCR-free survival compared to the positive SVI group. In addition, significant differences were observed in the prognosis of pathologic T3a and these two groups. This suggests that pathologic T3b can be stratified into two categories.

## Introduction

Seminal vesicle invasion (SVI) following radical prostatectomy (RP) is a well-known poor prognostic factor for prostate cancer (PCa)^1–3^. SVI is regarded to be associated with local relapse, distant metastasis, and early biochemical recurrence (BCR)^3^. However, according to the additional pathological features that have been reported by in-depth studies of RP specimens, patients with SVI have a relatively heterogeneous natural history after RP^4–9^. SVI occurs most commonly via extracapsular extension (ECE) into the soft tissues adjacent to the prostate and SV, then subsequently into the wall of the SV^9^. According to the route of spread, different SVI types have shown a different prognosis after RP^9,10^. However, preoperative clinical evaluation for PCa aggressiveness regarding with SVI remains elusive^5–8^.

In the past decade, a growing body of evidence has suggested the role of multi-parametric MRI (mpMRI) in the detection, risk stratification, and management of PCa^11–13^. With the wide use of mpMRI, several studies have also investigated the prognostic role of SVI on mpMRI^14,15^. Hegde *et al*.^14^ reported that mpMRI evidence of SVI was the only significant predictor of BCR (hazard ratio [HR], 13.98; P = 0.006) in patients with high-risk PCa treated with a combination of high-dose-rate brachytherapy and external beam radiotherapy. However, their study was limited by its small number of patients (n = 37) and retrospective study design. In addition, studies regarding pathological outcomes following RP are still lacking^15^.

Thus, the aim of the present study was to evaluate the prognostic role of SVI on preoperative mpMRI in pathological T3b PCa patients at RP. We also performed a comparative analysis between pathologic T3a and T3b patients according to the status of SVI on mpMRI.

## Materials and Methods

### Ethics statement

The Institutional Review Board of Seoul National University Bundang Hospital approved this study (Approval number: B-1706/402-115). A written informed consent of patients was waived by the Institutional Review Board as this was a retrospective study. Personal identifiers were completely deleted such that data were analyzed anonymously. Our study was conducted according to the ethical standards of the 1964 Declaration of Helsinki and its later amendments.

### Study cohort

We retrospectively reviewed the clinical data of patients who underwent RP and preoperative multiparametric prostate MRI (mpMRI) for clinically localized or locally advanced PCa between March 2008 and April 2018 at our institution. Patients who received neoadjuvant or adjuvant therapies (radiation and/or androgen deprivation therapy) were excluded. A total of 159 patients with pT3b PCa were stratified into the two groups based on the mpMRI findings (negative vs. positive SVI). A positive SVI was defined as the presence of mpMRI evidence of SVI. In addition, 290 patients with pT3a were also included in this study for further comparative analysis.

### Preoperative mpMRI protocol and image interpretation

All preoperative mpMRIs were performed after biopsy (usually two to six weeks later) using a 1.5- or 3-T system (Achieva Tx and Ingenia; Philips, the Netherlands) with a phase-array cardiac six-channel coil without using an endorectal coil. mpMRI consisted of axial T2-weighted imaging (T2WI), T1/T2-weighted registered imaging (T1/T2RI), diffusion-weighted imaging (DWI) with corresponding apparent-diffusion coefficient (ADC) maps, and dynamic contrast enhanced (DCE). The protocols were described in detail in our previous reports^16–18^ and appendix. All of the images were reviewed by two high-volume radiologists (H.J.L. and S.I.H.) with >20 years of experience interpreting prostate MRI using a Picture Archiving and Communication Systems workstation (PACS, INFINITT Technology, Seoul, Korea). The features of a positive SVI included focal or diffuse low signal intensity in T2WI and/or abnormal enhancement within the seminal vesicle in DCE, restricted diffusion, obliteration of the angle between the base of the prostate and the seminal vesicle, and demonstration of direct tumor extension from the base of the prostate into and around the seminal vesicle.

### Data acquisition and definitions

RPs were conducted by several surgeons using open, laparoscopic, or robotic modality. All pathological specimens were evaluated by a staff pathologist (G.C.) with genitourinary expertise. The following variables were compared between the categorical groups: age; body mass index (BMI); pre-biopsy prostate-specific antigen (PSA) level; pathologic Gleason score (GS); pathologic characteristics including extracapsular extension (ECE), positive surgical margin (PSM), and lymph node invasion (LNI); radiologic findings, including ECE and lymph node enlargement (LNE); and BCR. BCR was defined as two consecutive rises in PSA, with the last PSA 0.2 ng/ml or higher after the RP^19^.

### Statistical analyses

Comparative analyses of the clinicopathological characteristics between the negative and positive SVI groups were conducted using the Chi-squared test for categorical variables and either the independent t-test or Mann-Whitney U test for continuous variables. In addition, Kaplan-Meier survival analysis was used to calculate the survival estimates for BCR-free survival. Further, the log-rank test was used to conduct comparisons between the groups. We also conducted univariate and multivariate Cox-proportional hazard regression analyses to evaluate the significant variables associated with BCR. All statistical analyses were performed using IBM SPSS Statistics ver. 22.0 (Armonk, NY, USA), statistical package for R, ver. 2.13.2 (R Foundation for Statistical Computing [http://www.r-project.org/]). Statistical significance was considered in cases with a two-sided p value less than 0.05.

## Results

### Baseline characteristics

The mean patient age and follow-up were 67.0 ± 7.3 years and 42.0 (interquartile range, 18.0–64.0) months, respectively. In total, 52 patients (32.7%) had a positive SVI on preoperative mpMRI, and were thus stratified as the positive SVI group. The baseline characteristics of the negative and positive SVI groups are summarized in Table 1. There were significant differences between the two groups in pre-biopsy PSA (mean, 26.6 [negative SVI] vs. 62.5 [positive SVI], P = 0.009), RP GS category (P = 0.041), pathologic PSM (P < 0.001), positive ECE (P < 0.001), and LNE (P = 0.020) on preoperative mpMRI. However, there were no significant differences in the other variables, including age, BMI, pathologic ECE, and LNI. BCR occurred in 45 (28.3%) patients in total, with 25 (23.4%) cases in the negative SVI group and 20 (38.5%) cases in the positive SVI group.

**Table 1:** Baseline characteristics of all patients with pathologic T3b after RP (Total N = 159).

N (%) or Mean ± SD	Negative SVI on MRI (N = 107)	Positive SVI on MRI (N = 52)	P
Age, years	67.0 ± 7.2	66.9 ± 7.7	0.951
BMI, kg/m^2^	24.6 ± 3.0	24.9 ± 3.3	0.556
Pre-biopsy PSA, ng/ml	26.6 ± 29.7	62.5 ± 92.5	0.009
RP GS category			0.041
3 + 4	7 (6.5%)	1 (1.9%)	
4 + 3	50 (46.7%)	16 (30.8%)	
≥4 + 4	50 (46.7%)	35 (67.3%)	
Pathologic ECE, yes	88 (82.2%)	48 (92.3%)	0.099
Pathologic LNI, yes	21 (21.4%)	18 (34.6%)	0.080
Pathologic PSM, yes	43 (40.2%)	40 (76.9%)	<0.001
Positive ECE on MRI	41 (38.3%)	42 (80.8%)	<0.001
Positive LNE on MRI	16 (15.0%)	16 (30.8%)	0.020
BCR, yes	25 (23.4%)	20 (38.5%)	0.047

BCR, biochemical recurrence; BMI, body mass index; DM, diabetes mellitus; ECE, extracapsular extension; GS, Gleason score; HTN, hypertension; LNE, lymph node enlargement; LNI, lymph node invasion; MRI, magnetic resonance imaging; PSA, prostate-specific antigen; PSM, positive surgical margin; RP, radical prostatectomy; SD, standard deviation; SVI, seminal vesicle invasion.

### Survival outcomes and Cox-proportional hazard regression analyses

**Figure 1:**
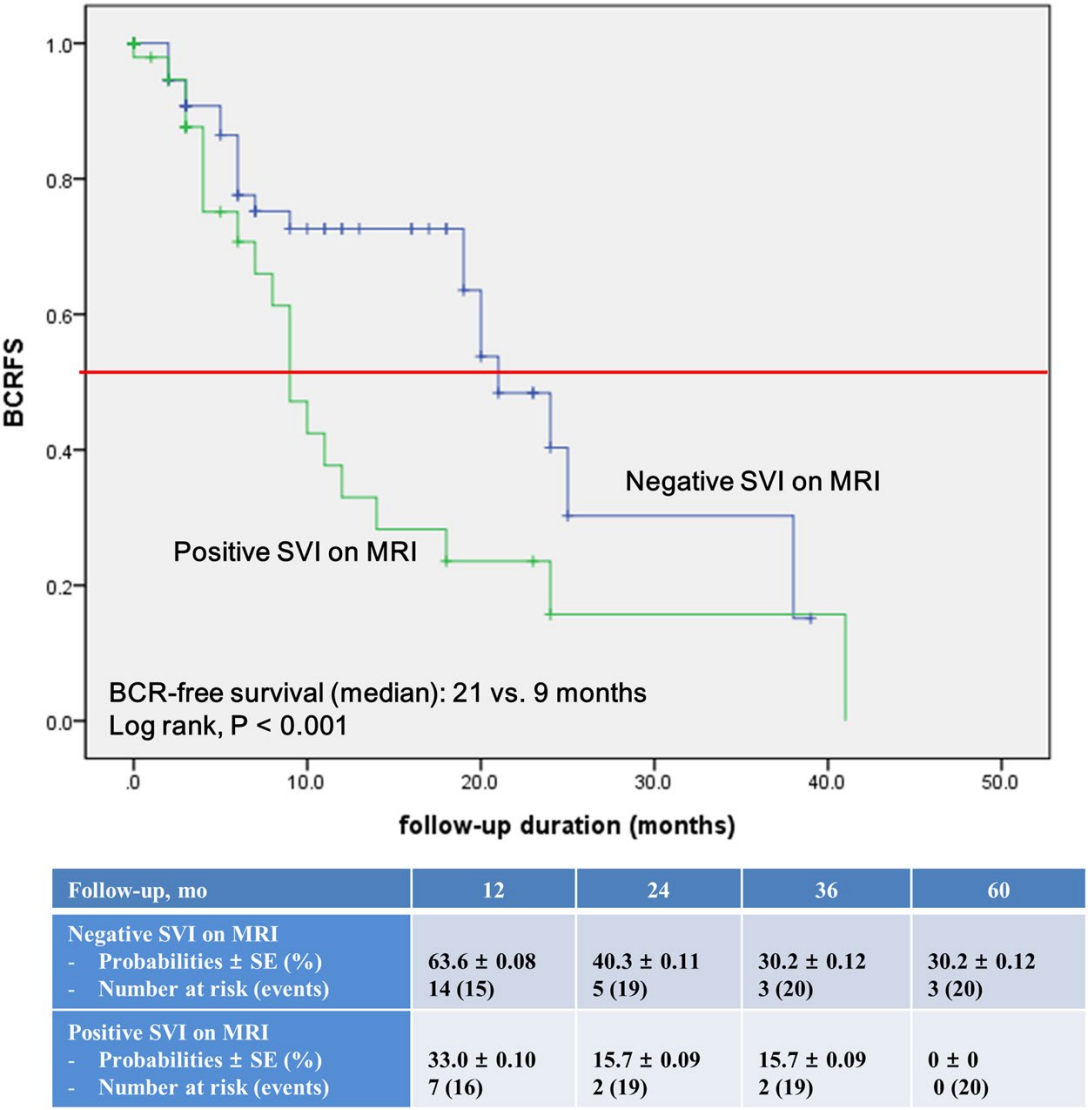
Kaplan–Meier estimate of biochemical recurrence-free survival following radical prostatectomy in patients with seminal vesicle invasion (SVI), stratified based on negative or positive SVI on magnetic resonance imaging.

Kaplan-Meier survival analysis in the two groups revealed significantly decreased BCR-free survival in the positive SVI group (median, 21 vs. 9 months, log-rank test, P < 0.001; Fig. 1).

Table 2 summarizes the results of univariate and multivariate Cox regression analyses to evaluate the variables associated with BCR in patients with pathologic T3b following RP. Univariate analysis revealed that pre-biopsy PSA (P < 0.001), RP GS category (P < 0.001), pathologic LNI (P = 0.017), pathologic PSM (P = 0.001), and positive SVI on preoperative mpMRI (P = 0.020) were significant predictors of BCR. On multivariate analysis, pre-biopsy PSA (P = 0.035) and positive SVI on preoperative mpMRI (P = 0.049) were identified as significant predictors of BCR.

**Table 2:** Univariate and multivariate Cox-proportional hazard regression analyses for BCR in patients with pathologic T3b after RP.

Variables	Univariate			Multivariate		
	HR	95% CI	P	HR	95% CI	P
Age	1.012	0.979–1.046	0.478			
BMI	1.066	0.964–1.179	0.212			
Pre-biopsy PSA	1.011	1.007–1.016	<0.001	1.006	1.000–1.011	0.035
RP GS category						
≤4 + 3	Reference					
≥4 + 4	3.191	2.021–5.037	<0.001	1.461	0.711–3.004	0.302
Pathologic ECE	0.596	0.258–1.380	0.227			
Pathologic LNI	2.109	1.142–3.894	0.017	0.724	0.313–1.675	0.451
Pathologic PSM	2.115	1.340–3.337	0.001	0.834	0.412–1.687	0.613
Positive SVI on MRI	2.101	1.126–3.924	0.020	1.913	1.003–3.648	0.049

BCR, biochemical recurrence; ECE, extracapsular extension; GS, Gleason score; MRI, magnetic resonance imaging; PSA, prostate-specific antigen; PSM, positive surgical margin; RP, radical prostatectomy; SVI, seminal vesicle invasion.

### Comparative analysis between pathologic T3a and T3b patients according to the SVI status on preoperative mpMRI

The baseline characteristics of the groups, including the pathologic T3a group, are summarized in Table 3. There were significant differences between the groups in pre-biopsy PSA, RP GS category, pathologic LNI, pathologic PSM (all, P < 0.001)

**Table 3:** Comparative analysis between pathologic T3a and T3b patients according to SVI status on MRI.

N (%) or Mean ± SD	pT3a (N = 290)	pT3b (N = 159)		P
		Negative SVI on MRI (N = 107)	Positive SVI on MRI (N = 52)	
Age, years	67.4 ± 7.0	67.0 ± 7.2	66.9 ± 7.7	0.857
BMI, kg/m^2^	24.7 ± 3.1	24.6 ± 3.0	24.9 ± 3.3	0.831
Pre-biopsy PSA, ng/ml	13.4 ± 11.3	26.6 ± 29.7	62.5 ± 92.5	<0.001
RP GS category				<0.001
3 + 3	1 (0.3%)	0 (0)	0 (0)	
3 + 4	78 (26.9%)	7 (6.5%)	1 (1.9%)	
4 + 3	133 (45.9%)	50 (46.7%)	16 (30.8%)	
≥4 + 4	78 (26.9%)	50 (46.7%)	35 (67.3%)	
Pathologic LNI, yes	7 (2.4%)	21 (21.4%)	18 (34.6%)	
Pathologic PSM, yes	112 (38.6%)	43 (40.2%)	40 (76.9%)	<0.001
BCR, yes	37 (12.8%)	25 (23.4%)	20 (38.5%)	<0.001

BCR, biochemical recurrence; BMI, body mass index; GS, Gleason score; LNI, lymph node invasion; MRI, magnetic resonance imaging; PSA, prostate-specific antigen; PSM, positive surgical margin; RP, radical prostatectomy; SVI, seminal vesicle invasion.

**Figure 2:**
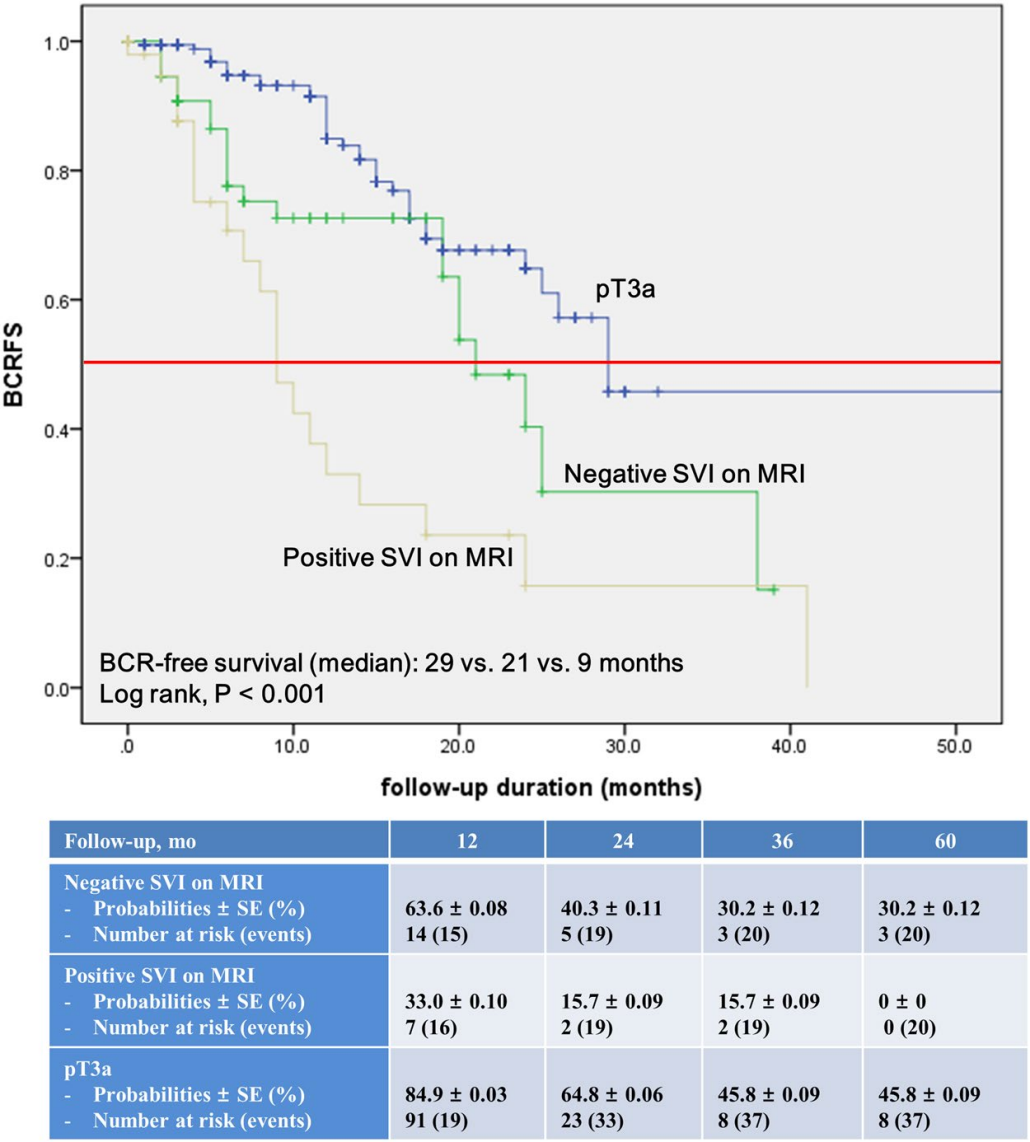
Kaplan–Meier estimate of biochemical recurrence-free survival following radical prostatectomy in patients with extracapsular extension or seminal vesicle invasion (SVI), stratified based on negative or positive SVI on magnetic resonance imaging.

Further comparative analysis with pathologic T3a group yielded significant differences between the groups throughout the Kaplan-Meier curve with decreased BCR-free survival in the positive SVI group (median, 29 vs. 21 vs. 9 months, log-rank test, P < 0.001; Fig. 2). In the subgroup of patients with pathologic GS ≥ 4 + 3, Kaplan-Meier survival analysis showed results consistent with those of the total cohort (median, 29 vs. 21 vs. 9 months, log-rank test, P < 0.001; Supplemental Fig. 1).

## Discussion

In the past decade, there has been a growing body of evidence suggesting a role of mpMRI in the detection and management of PCa^14^. A recent meta-analysis showed that, even with low sensitivity (58%), the specificity of mpMRI is reliable for SVI detection (96%)^20^. Accordingly, the guideline jointly revised by the European Association of Urology, the European Society for Radiotherapy and Oncology, and the International Society of Geriatric Oncology has recommended that mpMRI can be used in high-risk localized and locally advanced PCa, as detecting SVI can affect further treatment decisions^21^. In current study, among a total of 1403 patients who performed preoperative mpMRI, we also found that the sensitivity and specificity for corresponding pathological SVI was 45.4% and 95.3%, respectively.

Several studies have proposed mpMRI to identify patients at a higher risk of adverse pathology, in addition to conventional clinical parameters^22,23^. Gandaglia *et al*.^22^ reported that the combination of mpMRI with accurate biopsy information on MRI-targeted and systematic biopsies improves the accuracy of multivariable models based on clinical and mpMRI data alone. In addition, by integrating clinical data with mpMRI information, Martini *et al*.^23^ developed a simple model with the low number of variables. They reported that the model performs better at predicting SVI than relying on mpMRI alone; model could be a tool to estimate side-specific SVI. At this point of view, preoperative prediction of SVI is of paramount importance, both in terms of prognosis and surgical strategy (e.g. seminal vesicle sparing techniques vs. wide excision).

The RP pathologic information allows for refined clinical decision-making and patient counseling regarding the need for further adjuvant therapies. Along with established adverse pathologic features such as GS ≥ 8 and LNI, SVI has also been found to be independently associated with a poor prognosis^24–27^. However, the five-year BCR rates for patients with SVI were extremely variable, ranging from 8% to 68%. Previous studies have also reported that BCR in these patients depends on the concomitant presence of Gleason > 7 (9.9–40%), PSA > 10–25 (16–44%), and PSM (32.7–68%)^20,21,24^. Given this substantial variability in SVI, elucidating the predictors of adverse survival outcomes is crucial.

Despite the well-established risk of BCR, there has been no definitive consensus regarding the role of adjuvant and/or salvage radiation therapy (RT) in patients with SVI. External beam radiation therapy (EBRT) has traditionally been used in cases of adverse pathologic outcomes in order to maximize local control following RP. To date, there have been three landmark studies reporting on the utility of adjuvant RT in pathologic T3 PCa: European Organization for Research and Treatment of Cancer (EORTC) 22911^28^, Southwest Oncology Group (SWOG) 8794^29^, and ARO96-02^30^. The SWOG 8794 trial demonstrated metastasis-free and overall survival benefits in all patients with pT3 disease treated with immediate adjuvant RT^27^. By contrast, the EORTC 2291122 and the ARO96-02 trials failed to demonstrate a survival benefit^28,30^. However, all of these trials showed some benefit for BCR^28–30^. In terms of salvage RT, several previous studies also showed an improvement in the BCR rate (10–38%) in patients with SVI^31,32^. Nonetheless, the real outcome and benefit of adjuvant and/or salvage RT, particularly for patients with SVI, remain elusive due to the lack of guidelines for optimal timing and duration^28–33^. Thus, reliable preoperative assessment of aggressiveness is critical for ensuring the appropriate treatment decision is reached in individual patients with SVI. In cases of patients with more aggressive PCa, there would be no actual benefit to expect from local adjuvant and/or salvage RT with a worse prognosis in any case.

In the current study, the positive SVI group showed significantly decreased BCR-free survival compared with the negative SVI group (Fig. 1). On multivariate analysis, positive SVI on preoperative mpMRI was identified as a significant predictor of BCR (Table 2). In subgroup analysis to control the confounding factors, including patients who had pathologic GS ≥ 4 + 3, the positive SVI group still showed significantly decreased BCR-free survival (Supplemental Fig. 1). Accordingly, we would tentatively suggest that a trade-off between the side-effects of adjuvant RT and the prevention of disease progression can be made with careful consideration in patients with positive SVI on preoperative mpMRI. Further well-designed studies are warranted to answer this important and clinically relevant question.

The current study has some limitations. First, even as a study of large tertiary institution, the retrospective study design was a crucial drawback. Due to the lack of institutional standardized protocol (optimal timing and duration) for the adjuvant therapies, we excluded patients who received neoadjuvant or adjuvant therapies (RT and/or androgen deprivation therapy) in order to control for confounders. Consequently, we could not evaluate the results of adjuvant treatments. Second, we did not conduct a re-review of pathologic slides. Accordingly, the potential subsequent misclassification of some lesions might have affected outcomes. Third, the small sample size might limit the power of the study to detect predictors for BCR beyond mpMRI evidence of SVI. Finally, due to the rapid evolution of prostate MRI technology over a last decade (such as quantitative DWI/DCE, intravoxel incoherent motion, diffusion tensor imaging, diffusional kurtosis imaging, restriction spectrum imaging, radiomics analysis, hybrid positron emission tomography/MRI), we could not unify the MRI protocol during the study period. Accordingly, these might affect the performance of mpMRI. Therefore, an evaluation in a larger, prospective cohort would help validate these preliminary findings.

## Conclusions

The present study revealed a significant difference between the negative and positive SVI groups in terms of BCR-free survival in pathological T3b PCa patients. The unpredicted (negative) SVI group showed a favorable BCR-free survival compared to the positive SVI group. In addition, significant differences were observed in the prognosis of pathologic T3a and these two groups. This suggests that pathologic T3b can be stratified into two categories.

## Supplementary information

Supplementary information.
