# Supplementary material for: Prognostic value of seminal vesicle invasion on preoperative multi-parametric magnetic resonance imaging in pathological stage T3b prostate cancer
Source: Sci Rep. 2020 Mar 30;10:5693. doi: 10.1038/s41598-020-62808-z (PMC7105461; doi:10.1038/s41598-020-62808-z)
Supplement: Supplementary file 1 — Supplementary information. [file 41598_2020_62808_MOESM1_ESM.pdf]

TITLE

Prognostic value of seminal vesicle invasion on preoperative multi-parametric magnetic resonance imaging in pathological stage T3b prostate cancer

RUNNING HEAD

Prognostic value of SVI on mpMRI

AUTHORS AND INSTITUTIONS

Jung Kwon Kim[1], Hak Jong Lee[2], Sung Il Hwang[2], Gheeyoung Choe[3], Sung Kyu Hong[1,4*]

[1]Department of Urology, Seoul National University Bundang Hospital, Seongnam, Korea

[2]Department of Radiology, Seoul National University Bundang Hospital, Seongnam, Korea

[3]Department of Pathology, Seoul National University Bundang Hospital, Seongnam, Korea

[4]Department of Urology, Seoul National University College of Medicine, Seoul, Korea

CORRESPONDENCE*

Sung Kyu Hong, MD, PhD.

Professor; Department of Urology, Seoul National University College of Medicine, Seoul, Korea; Seoul National University Bundang Hospital, Seongnam, Korea 173-82, Gumi-Ro, Bundang-gu, Seongnam-si, Gyeonggi-do, 13620, Korea

Tel: 82-31-787-7343, Fax: 82-31-787-4057, E-mail: skhong@snubh.org

Supplemental Figure 1. Subgroup of patients with pathologic GS ≥ 4+3: Kaplan–Meier estimate of biochemical recurrence-free survival after radical prostatectomy in patients with extracapsular extension or seminal vesicle invasion (SVI), stratified by the negative or positive of SVI on magnetic resonance imaging.

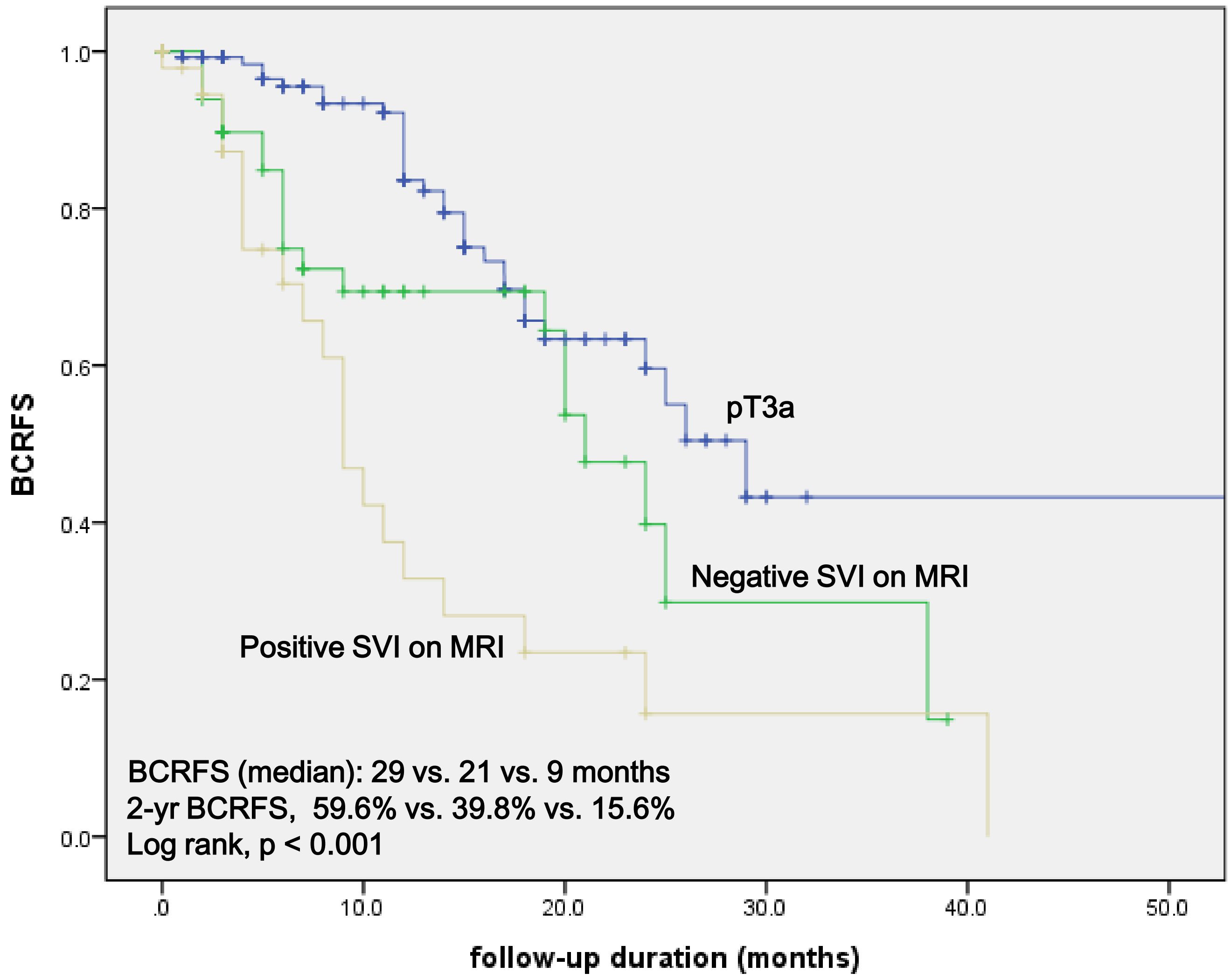

APPENDIX

The detailed parameters of the T2 weighted MR were as follows: TR, 2500–3000ms; TE, 70–90 ms; slice thickness, 3 mm; inter slice gap,1 mm; field of view, 160 mm × 160 mm; matrix, 320 × 320; and number of excitations, 1. Diffusion encoding gradients were applied as a bipolar pair at b-values 0 and 1000 s/mm^2. Apparent diffusion coefficient (ADC) maps were automatically generated on a pixel-by-pixel basis.
